# Changes in health-related quality of life and its influencing factors of patients after percutaneous coronary intervention in China: A single-center longitudinal study

**DOI:** 10.1097/MD.0000000000046428

**Published:** 2026-05-12

**Authors:** Qiang Fu, Lei Dou, Zhuxin Mao, Shunping Li

**Affiliations:** a Department of Cardiovascular Surgery, General Hospital of Tianjin Medical University, Tianjin, China; b Department of Social Medicine and Health Management, School of Public Health, Cheeloo College of Medicine, Shandong University, Jinan, Shandong, China; c NHC Key Lab of Health Economics and Policy Research (Shandong University), Jinan, China; d Center for Health Management and Policy Research, Shandong University (Shandong Provincial Key New Think Tank), Jinan, Shandong, China; e Center for Health Preference Research, Shandong University, Jinan, China; f Centre for Health Economics Research and Modelling Infectious Diseases, Vaccine and Infectious Disease Institute, University of Antwerp, Belgium.

**Keywords:** coronary heart disease, EQ-5D-5L, health-related quality of life, percutaneous coronary intervention, Seattle Angina Questionnaire

## Abstract

This study aimed to evaluate changes in health-related quality of life (HRQoL) of patients within 4 weeks after percutaneous coronary intervention (PCI) using generic and disease-specific instruments and to explore its influencing factors. In this prospective, longitudinal study, 79 patients with coronary heart disease were recruited from the Cardiology Department of the General Hospital of the Tianjin Medical University, China. The EQ-5D-5L and Seattle Angina Questionnaire (SAQ) were used to evaluate patients’ HRQoL at baseline, and 1 and 4 weeks after PCI. Tobit regression model and multiple linear regression were used to analyze factors associated with patients’ HRQoL changes. A final validated sample of 75 patients was analyzed. The EQ-5D-5L health state utility score improved from 0.85 (standard deviation [SD]: 0.15) at baseline to 0.95 (SD: 0.11) and 0.98 (SD: 0.10) at 1 and 4 weeks after PCI, respectively. Pain/discomfort and angina stability were the most improved dimensions on the EQ-5D-5L and SAQ, respectively. Patients who were retired, had high subjective well-being, and had high baseline HRQoL scores showed greater improvement in EQ-5D-5L scores after PCI, while the baseline HRQoL scores were positively associated with SAQ score improvement. A greater benefit from PCI was observed among coronary heart disease patients within 4 weeks after this procedure. More attention in clinical care should be provided to retired patients, those with low subjective well-being, and those with a low baseline HRQoL.

## 1. Introduction

Coronary heart disease (CHD), one of the most common cardiovascular diseases, represents a major public health concern worldwide. Along with stroke, CHD accounts for the largest number of premature cardiovascular diseases-related deaths.^[1]^ By prediction, CHD will be responsible for 30.5% of the global death toll by 2030.^[2]^ By estimate, more than 11 million people in China live with CHD, of which the morbidity will increase steadily over the next few decades.^[3]^ The mortality rates of CHD were 121.59 per 100,000 people in urban areas and 130.14 per 100,000 people in rural areas of China in 2019, and these numbers continue to rise.^[4]^ CHD treatments include therapeutic medications, percutaneous coronary intervention (PCI), and coronary artery bypass grafting (CABG).^[5]^ A growing body of evidence indicates that PCI is more effective, safer, less disabling, and less expensive than CABG.^[6]^ Considered a breakthrough method, PCI is frequently used as a revascularisation strategy in CHD patients.^[7]^ In 2018, the total number of PCI for CHD in mainland China was 915,256, and the growth rate of PCI cases was 21.5%.^[8]^

Patients undergo a spectrum of long-term treatment after PCI, which affects multiple aspects of their lives, including physical, mental, and social health.^[9]^ There is a growing recognition of the importance of maintaining or improving patients’ health-related quality of life (HRQoL) in addition to traditional outcome measures, such as death and myocardial infarction.^[10]^ As a multidimensional concept, HRQoL refers to an individual’s capacity to perform daily activities (i.e., functioning), in addition to their life perspective (i.e., well-being) and subjective management of their health condition.^[11]^ HRQoL has gained attention in recent years as an important patient-reported outcome measure to inform patient-centered care, clinical decision-making, and health policy or reimbursement decisions.^[12]^

A large variety of generic and disease-specific instruments have been developed to assess the HRQoL in CHD patients.^[13]^ Since many CHD patients have comorbidities, optimal assessment may be achieved by using both generic and disease-specific HRQoL instruments.^[1]^ For cost-effectiveness assessment, cost-utility analyses have been increasingly conducted to support decision makers in the allocation of scarce healthcare resources. Generic preference-based instruments can provide data for comparison across different patient groups and generate health state utility (HSU) scores, which are applied to calculate quality-adjusted life years for cost-utility analyses.^[14]^ Disease-specific instruments are more likely to capture the effects of specific symptoms attributable to the condition, such as the frequency and intensity of angina during PCI.^[15]^

Several studies have examined HRQoL changes of CHD patients after PCI. However, few studies have used generic and disease-specific instruments to comprehensively capture different aspects of HRQoL. Moreover, inconsistent findings on the short-term impact of PCI on patient’s HRQoL have been observed. Some studies demonstrated that patients had major adverse cardiovascular events and high anxiety and depression scores in the 1st month after PCI,^[16,17]^ while others showed improved HRQoL, particularly with important improvements in angina and physical function, for all patients following PCI.^[18]^ An important knowledge gap remains in the evaluation of short-term HRQoL benefits of PCI. Additionally, while previous studies have investigated factors influencing HRQoL after PCI, such as sex, age, anxiety, depressive symptoms, number of diseased vessels, comorbidities,^[19,20]^ evidence related to this topic is scarce in China. Therefore, it is essential to identify the associated factors, particularly modifiable ones, to further develop evidence-based health promotion and intervention programs in China. This study aimed to compare HRQoL changes of patients within 4 weeks after PCI and investigate factors associated with HRQoL changes.

## 2. Methods

### 2.1. Study design and population

In this prospective, single-center longitudinal study, data were collected at the General Hospital of the Tianjin Medical University, China, between April and September 2019. The inclusion criteria for patients were as follows: cardiology inpatients aged 18 years or older who had been clearly diagnosed with CHD and were scheduled to undergo PCI as part of their clinical treatment for CHD, with the PCI performed 1 or 2 days after hospitalization. The exclusion criteria were unwillingness to provide informed consent, inability to understand the questionnaires, serious comorbidities (such as malignant tumors), history of mental illness, or hearing or vision impairment. Ethical approval was obtained from the Ethics Review Board of the School of Health Care Management, Shandong University (reference number: ECSHCMSDU20191002), and the study was conducted in adherence to the tenets of the Declaration of Helsinki.

Informed consent was obtained from all participants before study enrollment. After the patients were hospitalized but before they underwent PCI surgery, well-trained medical staff conducted the baseline survey (T0), which included sociodemographic information, clinical information, and HRQoL assessment. Patients were contacted by the same medical staff over telephone in the 1st week (T1) and 4th weeks (T2) after PCI to reassess their HRQoL.

Sample size calculation formula for single-group repeated measures was used, $N=\frac{2{\left(\mu_{1-\alpha /2}+\mu_{1-\beta }\right)}^{2}S^{2}[\left(1+\left(n-1\right)\rho \right]}{n{\left(\mu_{1}-\mu_{2}\right)}^{2}}$^[21]^ with α =0.05, μ_1-α⁄2_ = 1.96, β = 0.20, μ_β_ = 0.84. Here, n represents the number of repeated measurements, and n = 3 in this study. Based on previous research^[22]^ to set ρ at 0.45, μ_1_ − μ_2_ at 0.09, and *s*^2^ at 0.05, the calculated sample size was 44. Considering a dropout rate of 30%, a minimum of 63 participants would be required.

Based on previous literature reviews^[18,23]^ and consultation with clinical experts, sociodemographic characteristics included age, sex, marital status, education level, occupation, monthly income, and lifestyle. Clinical characteristics included CHD type, duration, disease state, and comorbidities, which were recorded by the patient’s attending doctor.

**Figure 1.**
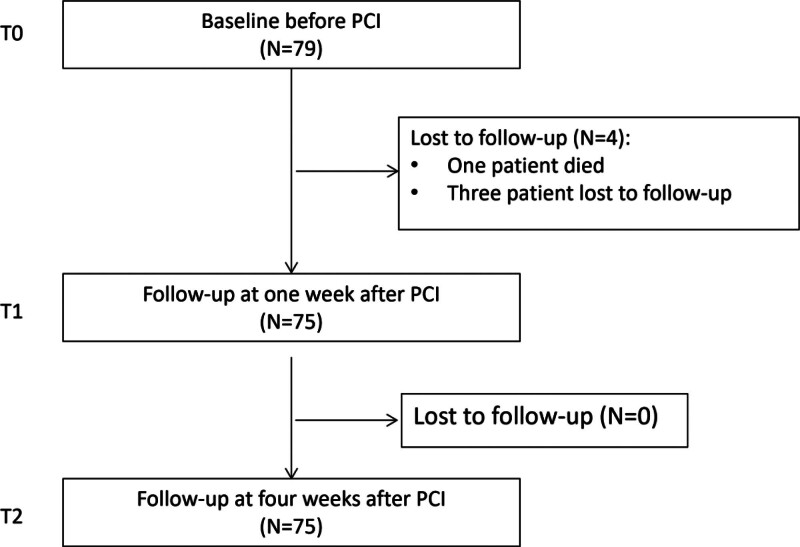
Flowchart of CHD patients from baseline to follow-up after PCI. CHD = coronary heart disease, PCI = percutaneous coronary intervention.

Patients completed the preoperative HRQoL questionnaire (T0: EQ-5D-5L and Seattle Angina Questionnaire [SAQ]) in the hospital before PCI and the second questionnaire (T1: EQ-5D-5L) at 1 week after PCI. SAQ was not included at T1, as it measures HRQoL changes over the past 4 weeks. The final questionnaire (T2: EQ-5D-5L and SAQ) was completed at 4 weeks after PCI. In total, 79 patients were eligible for inclusion in the baseline study. Within 1 week after PCI, 4 patients were lost to follow-up, including 1 death and 3 who could not be contacted. The final data used for the analysis were collected from 75 patients who completed the surveys at all 3 assessment points (Fig. 1).

### 2.2. HRQoL measurement

The EQ-5D-5L is a generic, preference-based instrument, which includes a descriptive health system based on 5 dimensions. The dimensions cover mobility (MO), self-care (SC), usual activities (UA), pain/discomfort (PD), and anxiety/depression (AD), and are characterized by 5 levels of response (no problems, slight problems, moderate problems, severe problems, and extreme problems).^[24]^ The Chinese version of the EQ-5D-5L descriptive system and Chinese-specific scoring algorithm of the EQ-5D-5L were adopted in this study.^[25]^ The score ranges from −0.391 to 1 (1: full health, 0: death), and a score < 0 represents a health status worse than death.

The SAQ is a 19-item disease-specific questionnaire that measures health status of CHD patients across 5 domains: physical limitation (PL), angina stability (AS), angina frequency (AF), treatment satisfaction, and disease perception. AS is measured by the prevalence of chest pain with strenuous activity, whereas AF reflects the number of times a person experiences chest pain and receives treatment for the symptoms with nitroglycerin.^[26]^ All domain and summary scores were transformed to a range of 0 to 100 by subtracting the lowest possible score, dividing the scale range, and multiplying by 100.^[27]^ Higher scores indicate less angina, fewer PL, and better HRQoL. The Chinese version of SAQ has been shown to be a valid, responsive, and reliable instrument.^[28]^

The World Health Organisation-Five Well-Being Index (WHO-5) is one of the most widely used generic questionnaires for subjective well-being assessment. WHO-5 consists of 5 positive items related to positive mood (good spirit and relaxation), vitality (being active and waking up fresh and rested), and general interest (being interested in things). Each item is scored from 5 (all the time) to 0 (none of the time), and the raw scores has a theoretical range of 0 (absence of well-being) to 25 (maximal well-being). A score < 13, which signals poor well-being, is an indication for depression testing under the International Classification of Diseases-10. WHO-5 has been applied to a wide range of diseases or conditions.^[29]^ The Chinese version of the WHO-5 has good validity and reliability.^[30]^

### 2.3. Statistical analysis

Means and standard deviations (SD) were used to calculate the EQ-5D-5L HSU and SAQ scores, while frequencies and percentages were used for categorical variables and for reporting any problems in each EQ-5D-5L dimension. Owing to the skewed distribution of both the EQ-5D-5L HSU and SAQ scores, nonparametric tests were used to analyze these data. Differences in the mean EQ-5D-5L scores at baseline, and 1 and 4 weeks after PCI among the entire cohort were tested using the paired Friedman test. Differences in the mean SAQ scores between the baseline time point and at 4 weeks after PCI were tested using the paired Wilcoxon signed-rank test.

HRQoL change was defined as the difference between postoperative and baseline scores. As the EQ-5D-5L HSU scores were left-skewed with a large proportion of respondents in full health, a Tobit regression model was used, whereas a multiple linear regression was used to identify significant independent predictors for SAQ scores. For the analysis, potential predictors included sex (male, female), age (30–54, 55–64, ≥65 years), educational level (illiteracy or primary school, secondary school, high school or technical secondary school, university degree and higher), occupation (working, retired), marital status (married, unmarried), monthly income (<2000, 2000–4000, >4000 RMB), lifestyle (smoking, drinking, exercise), CHD duration (≤0.5, 0.51–1, >1 month), disease state (1st episode, relapse), types of comorbidity, WHO-5 scores (<13, ≥13), and baseline EQ-5D-5L HSU and SAQ scores. All data were analyzed using SPSS 24.0 (IBM Corporation, Armonk) and STATA 15.0 (Stata Corp, College Station), with statistical significance set at *P* < .05.

## 3. Results

### 3.1. Characteristics of the patients

Sociodemographic and clinical characteristics of the patients are presented in Table 1. Of 75 study participants, 47 (62.7%) were men, with the mean ± SD age of 64.6 ± 9.1 years. Most patients were retired (73.3%) and married (96.0 %). Half of them (50.7%) had a monthly income of more than 4000 RMB (approximately 620 United States Dollars). Most patients (86.7%) were diagnosed with unstable angina, the mean CHD duration was 1.6 ± 2.0 months, and 54.7% of patients were in the 1st episode. Many patients had comorbidities, including hypertension (65.3%), diabetes (37.3%), and hyperlipidemia (28.0%).

**Table 1:** Demographic and clinical characteristics of patients (n = 75).

Characteristics	N (%) or mean ± SD
Sociodemographic	
Gender	
Male	47 (62.7)
Female	28 (37.3)
Age (yr)	
Mean ± SD	64.6 ± 9.1
Range	39–84
Educational level	
Illiteracy or primary school	8 (10.7)
Secondary school	33 (44.0)
High school or technical secondary school	22 (29.3)
University degree and above	12 (16.0)
Occupation	
Working*	20 (26.7)
Retired	55 (73.3)
Marital status	
Married	72 (96.0)
Unmarried†	3 (4.0)
Monthly income (Chinese Yuan, CNY)	
≤4000	37 (49.3)
>4000	38 (50.7)
Smoking	
Yes	13 (17.3)
No	62 (82.7)
Drinking	
Yes	13 (17.3)
No	62 (82.7)
Exercise	
Yes	26 (34.7)
No	49 (65.3)
Clinical characteristics	
CHD type	
Stable angina	1 (1.3)
Unstable angina	65 (86.7)
Acute myocardial infarction	9 (12.0)
Duration of CHD (mo)	
Mean ± SD	1.6 ± 2.0
Range	0–12
Disease state	
First episode	41 (54.7)
Relapse	34 (45.3)
Prevalence of comorbidities	
Hypertension	49 (65.3)
Diabetes	28 (37.3)
Hyperlipidemia	21 (28.0)
Subjective well-being	
WHO-5 scores < 13	7 (9.3)
WHO-5 scores ≥ 13	68 (90.7)

* Working includes working in public institutions and companies, civil servants, and freelancers.

† Unmarried includes single, divorced, widowed, and separate.

### 3.2. Patients’ HRQoL changes across 3 time points

Table 2 shows HRQoL changes over time as assessed using the EQ-5D-5L and SAQ. A significant improvement was observed in both the EQ-5D-5L HSU and SAQ scores from baseline to 1 and 4 weeks after PCI. The EQ-5D-5L HSU score improved from 0.85 (SD: 0.15) at baseline to 0.95 (SD: 0.11) and 0.98 (SD: 0.10) at 1 and 4 weeks after PCI, respectively, (*P* < .001). Additionally, the mean SAQ summary score increased by 15.67 from 56.41 (SD: 10.15) at baseline to 72.08 (SD: 6.38) at 4 weeks after PCI (*P* < .001).

**Table 2:** Changes in HRQoL from baseline to 1 week and 4 weeks after PCI (n = 75).

HRQoL	Baseline (mean ± SD)	1 week after PCI† (mean ± SD)	4 weeks after PCI (mean ± SD)	*P*-value‡
EQ-5D-5L				
HSU score	0.85 ± 0.15	0.95 ± 0.11	0.98 ± 0.10	<.001*
SAQ				
Physical limitation	54.01 ± 11.97	N/A	63.20 ± 9.77	<.001*
Angina stability	26.67 ± 26.74	N/A	89.33 ± 17.52	<.001*
Angina frequency	59.73 ± 26.46	N/A	95.47 ± 10.17	<.001*
Treatment satisfaction	68.00 ± 9.44	N/A	77.96 ± 7.05	<.001*
Disease perception	51.44 ± 10.47	N/A	65.78 ± 6.06	<.001*
Summary score	56.41 ± 10.15	N/A	72.08 ± 6.38	<.001*

HRQoL = health-related quality of life, HSU = health state utility, PCI = percutaneous coronary intervention, SAQ = Seattle Angina Questionnaire.

† Due to 4-week recall of the SAQ, only EQ-5D-5L was completed at 1 week after PCI.

‡ Differences in mean scores of EQ-5D-5L in 3 time point were tested with the Friedman test (paired). Differences in scores of the SAQ were tested with the Wilcoxon signed-rank test (paired).

* *P* < .05.

**Figure 2.**
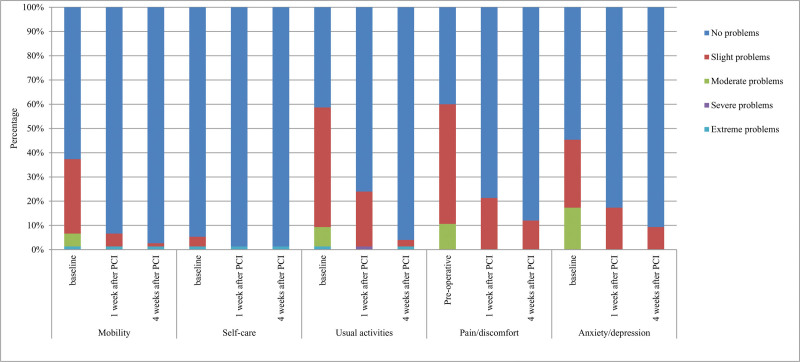
Temporal changes of health problems reported of patients in EQ-5D-5L.

**Figure 3.**
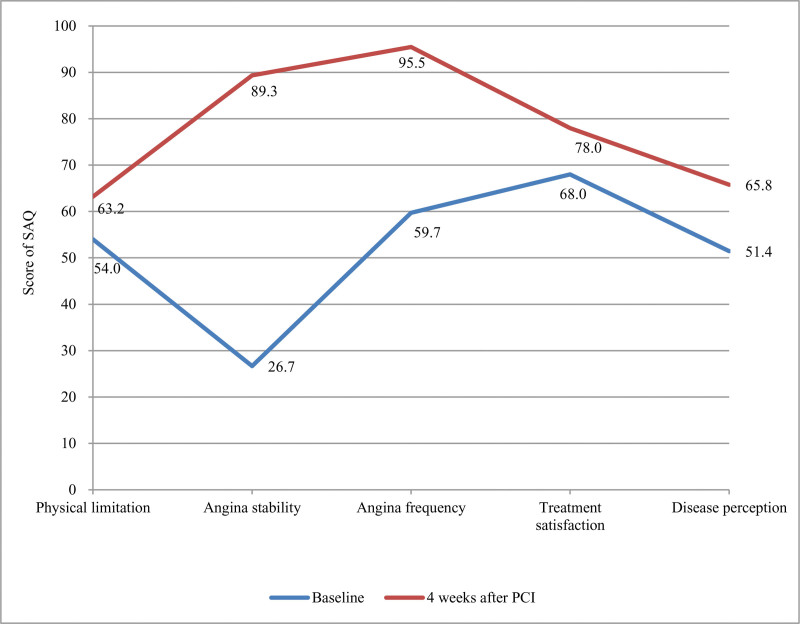
Temporal changes in each domain of the SAQ. SAQ = Seattle Angina Questionnaire.

There was a significant improvement in each dimension as compared to the baseline value among patients who reported problems. For EQ-5D-5L (Fig. 2), PD was the most frequently reported problem (60.0%), followed by UA (58.7%) at baseline, which were the 2 largest increased dimensions after PCI. The frequency of patients who reported PD decreased to 22.3% and 12.0% at 1 and 4 weeks after PCI, respectively. Similarly, the frequency of patients who reported impaired UA decreased to 24.0% and 4.0% at 1 and 4 weeks after PCI, respectively. Regarding the SAQ (Fig. 3), the most improved domain was AS, which increased from 26.7 at baseline to 89.3 at 4 weeks after PCI. More details on HSU changes according to patient characteristics at the 3 time points are provided in Table S1, Supplemental Digital Content, https://links.lww.com/MD/Q843.

### 3.3. Factors associated with HRQoL changes

The results of the multivariate analyses of associated factors with HRQoL changes are shown in Table 3. Factors significantly associated with HRQoL score changes were occupation, subjective well-being, and baseline HRQoL scores. Retired patients showed a greater improvement in the EQ-5D-5L HSU score changes than working patients (*P* = .002). Meanwhile, patients with better subjective well-being, as indicated by the WHO-5 scores, had a greater improvement in the EQ-5D-5L HSU score (*P* = .005). Baseline HRQoL, including EQ-5D-5L HSU and SAQ scores, was significantly associated with improvements in the EQ-5D-5L HSU and SAQ scores. In contrast, sex, age, monthly income, CHD duration, disease state, and comorbidities were not significantly associated with HRQoL changes.

**Table 3:** Predictors of changes of EQ-5D-5L HSU scores and SAQ scores.

Predictor variables	EQ-5D-5L HSU score changes			SAQ score changes		
	COE	SE	*P*-value	COE	SE	*P*-value
Gender (ref.=male)						
Female	0.022	0.023	.337	1.073	1.347	.429
Age (yr)	-0.002	0.001	.252	-0.068	0.090	.451
Educational level (ref. = illiteracy or primary school)						
Secondary school	-0.088	0.043	.047*	-4.778	2.631	.075
High school or technical secondary school	-0.087	0.044	.053	-3.001	2.738	.278
University degree and above	-0.059	0.048	.220	-2.412	2.888	.407
Occupation (ref. = working)						
Retired	0.110	0.034	.002*	2.807	2.160	.199
Marital status (ref. = married)						
Unmarried	0.045	0.060	.458	-0.612	3.527	.863
Monthly income (ref. = ≤4000)						
>4000	0.026	0.023	.264	0.620	1.365	.651
Smoking (ref. = Yes)						
No	-0.002	0.036	.960	-3.514	2.083	.097
Drinking (ref. = Yes)						
No	-0.029	0.037	.433	2.481	2.113	.246
Exercise (ref. = Yes)						
No	0.031	0.026	.235	2.623	1.541	.095
Duration of CHD (mo)	0.004	0.005	.452	0.027	0.314	.933
Disease state (ref. = First episode)						
Relapse	0.036	0.022	.110	-0.295	1.336	.826
Comorbidity with hypertension (ref. = Yes)						
No	-0.020	0.023	.379	1.610	1.345	.236
Comorbidity with diabetes (ref. = Yes)						
No	-0.015	0.023	.522	-1.220	1.368	.376
Comorbidity with hyperlipidemia (ref. = Yes)						
No	-0.031	0.023	.179	0.906	1.407	.522
Subjective well-being (ref. = WHO-5 < 13)						
WHO-5 ≥ 13	0.120	0.041	.005*	1.117	2.687	.679
Baseline EQ-5D-5L HSU score	-0.543	0.094	<.001*	-0.766	4.320	.047*
Baseline SAQ score	-0.003	0.001	.012*	-0.940	0.082	<.001*

CHD = coronary heart disease, COE = coefficient, HSU = health state utility, PCI = percutaneous coronary intervention, SAQ = Seattle Angina Questionnaire, SE = standard error.

* *P* < .05.

## 4. Discussion

In this study, we investigated HRQoL changes before and after PCI using generic and disease-specific instruments. Furthermore, we explored factors associated with changes in HRQoL. We observed significant improvements in HRQoL in patients within 1 and 4 weeks after PCI. Occupation, subjective well-being, and baseline HRQoL scores were associated with HRQoL score changes.

The mean EQ-5D-5L HSU scores at baseline, and 1 and 4 weeks after PCI were 0.85, 0.95, and 0.98, respectively, indicating that patients’ HSU is largely similar to the normative scores for the Chinese population 1 week after PCI and higher than the normative scores 4 weeks after PCI.^[31]^ The findings highlighted that patients’ HRQoL improvement followed the recovery trajectory. As previously reported, physical symptoms often improved in a steady linear direction after cardiac surgery.^[32]^ The results of this study suggested that regular assessments of the disease recovery trajectory should be included in patient management.

Patients at baseline reported problems in PD and UA and were observed to have lower AS and AF scores. Rapid improvements were observed after PCI, which is consistent with previous studies showing an incremental benefit of patients from PCI.^[33–35]^ The benefits of PCI on symptom relief, improved quality of life, improved left ventricular function, and avoidance of CABG and reduced arrhythmia have been confirmed.^[36,37]^ However, previous studies indicated that at 1 month after PCI, this procedure was significantly associated with better patient-reported scores for symptoms of angina, dyspnea, and depression than other therapies; however, these differences decreased at 12 months postoperatively and was no longer statistically significant at 36 months after surgery.^[38]^ This finding might provide an explanation for the fact that PCI does not change the pathological process of atherosclerosis and that stent will accompany the patient’s blood vessels for life. Patients may experience risk events, such as arrhythmia, stent restenosis, and thrombosis,^[39]^ which may lead to mental stress, anxiety, and impaired HRQoL.^[40]^ Therefore, it is important to consider the frequency and timing of HRQoL assessments to accurately determine treatment effects.

Regarding factors influencing HRQoL changes, occupation was associated with HRQoL improvement, and retired patients’ HRQoL improved to a larger extent than that of working patients. In addition to differences in patient characteristics (e.g., the mean ages of working vs retired patients were 58.3 years vs 66.8 years, respectively, in this study). Other notable reasons might explain for this difference. Available evidence indicates that older patients improve as much as younger patients, particularly in the areas of physical function and angina status.^[41]^ Additionally, older patients might have lower expectations of recovery from PCI, which could influence their perceptions of health to be markedly better than expected.^[42]^ Furthermore, working patients would have less time to recover from PCI than retirees since they had to return to work prematurely without adequate rest and postoperative rehabilitation,^[7]^ while retirees were able to focus on recovery to improve their HRQoL because of an increase in life satisfaction and a higher degree of autonomy due to sufficient financial capacity.^[43]^

Better subjective well-being (i.e., WHO-5 ≥ 13) was positively associated with patients’ HRQoL changes. Subjective well-being refers to a broad category of phenomena, including emotional responses, domain satisfaction, and global judgements of life satisfaction, which should be considered when quantifying an individual’s well-being.^[44]^ A growing body of evidence indicates that subjective well-being and health are closely related and positive subjective well-being is associated with better cardiovascular health.^[45]^ Other studies demonstrated that positive subjective well-being might directly affect heart disease via alterations in the neuroendocrine, cardiovascular, and inflammatory systems,^[46]^ and indirectly affect heart disease via health behaviors, such as improved diet and increased physical activity.^[47]^ According to several studies, depressive symptoms were commonly detected in patients during the post-myocardial infarction period,^[48]^ and high anxiety and depression scores in the 1st month after PCI were previously observed.^[16,49]^ Therefore, early detection and treatment of depression and improvement of the subjective well-being of CHD patients are crucial for improving a patient’s quality of life and possibly preventing a recurrent coronary event.

In this study, baseline HRQoL (EQ-5D-5L HSU and SAQ scores) was an important predictor of HRQoL changes after PCI. Most benefits were observed in patients with more severe and frequent angina at baseline, which is consistent with the results of previous studies.^[15,33]^ As previously reported, significant predictors of 1-year HRQoL improvement were the patients’ AF and PL before PCI.^[18]^ This observation is biologically plausible because myocardial ischemia with angina caused by CHD reduces functional capacity, leading to decreased activity levels and HRQoL impairment. PCI relieves angina, restores functional capacity, and improves HRQoL. These findings highlighted the importance of measuring baseline health status before PCI. Self-reported health status may be included as a potential screening tool, and individualized follow-up regimens may be planned according to the patients’ baseline HRQoL.

This study had some limitations. First, this was a single-center study with a relatively small sample size, which might have limited the generalisability of the results. Predictors of patients’ HRQoL improvement should be examined in larger multicentre studies. Second, some important predictors of the outcome, such as revascularisation, number of heart stents, procedural results, and post-procedural complications, were not measured, and future efforts to further improve these prediction models are warranted. Third, the study participants, recruited from the General Hospital of the Tianjin Medical University, was associated with older age, higher income, and an urban population, which might have led to more pronounced HRQoL benefits.

## 5. Conclusions

This study used generic and disease-specific HRQoL instruments simultaneously to measure HRQoL changes before and after PCI and found a significant improvement in HRQoL. Occupation, subjective well-being, and baseline HRQoL were associated with HRQoL changes in patients who underwent PCI. More attention in hospital-based clinical care should be provided to retired patients, those with low subjective well-being, and those with low baseline HRQoL. It is necessary to comprehensively consider clinical and technical factors and patients’ HRQoL when selecting PCI for CHD treatment.

## Acknowledgments

We acknowledge the Department of Cardiovascular surgery, General Hospital of Tianjin Medical University for supporting this study. Thanks to all the participants for the contribution to this study.

## Author contributions

**Conceptualization**: Qiang Fu, Lei Dou, Shunping Li.

**Data curation**: Qiang Fu, Zhuxin Mao, Shunping Li.

**Formal analysis**: Lei Dou, Zhuxin Mao.

**Funding acquisition**: Qiang Fu, Lei Dou.

**Investigation**: Qiang Fu, Shunping Li.

**Methodology**: Zhuxin Mao.

**Project administration**: Qiang Fu, Lei Dou, Shunping Li.

**Resources**: Qiang Fu, Lei Dou, Shunping Li.

**Software**: Qiang Fu, Lei Dou, Zhuxin Mao.

**Supervision**: Qiang Fu, Lei Dou, Shunping Li.

**Validation**: Shunping Li.

**Visualization**: Zhuxin Mao, Shunping Li.

**Writing – original draft**: Qiang Fu.

**Writing – review & editing**: Qiang Fu, Lei Dou, Zhuxin Mao, Shunping Li.

## Supplementary Material
