# Supplementary material for: Changes in health-related quality of life and its influencing factors of patients after percutaneous coronary intervention in China: A single-center longitudinal study
Source: Medicine (Baltimore). 2026 May 12;104(49):e46428. doi: 10.1097/MD.0000000000046428 (PMC12689147; doi:10.1097/MD.0000000000046428)
Supplement: Supplementary file 1 [file medi-104-e46428-s001.docx]

**Supplementary Table 1. Health state utility by characteristics of patients in different time-points**

| **Characteristics** | **Baseline**  **Mean (95%CI)** | ***P*-value** | **1 week after PCI**  **Mean (95%CI)** | ***P*-value** | **4 weeks after PCI**  **Mean (95%CI)** | ***P*-value** |
| --- | --- | --- | --- | --- | --- | --- |
| **Gender** |  | 0.737 |  | 0.938 |  | 0.648 |
| Male | 0.85 (0.80,0.90) |  | 0.95 (0.92,0.99) |  | 0.97 (0.93,1.00) |  |
| Female | 0.85 (0.80,0.90) |  | 0.96 (0.93,0.99) |  | 0.99 (0.97,1.00) |  |
| **Age (years)** |  | 0.799 |  | 0.210 |  | 0.223 |
| 30-54 | 0.84 (0.75,0.94) |  | 0.97 (0.92,1.00) |  | 1.00 (1.00,1.00) |  |
| 55-64 | 0.84 (0.77,0.91) |  | 0.95 (0.90,1.00) |  | 0.96 (0.90,1.00) |  |
| ≥ 65 | 0.86 (0.82,0.90) |  | 0.95 (0.93,0.98) |  | 0.98 (0.97,1.00) |  |
| **Educational level** |  | 0.542 |  | 0.256 |  | 0.583 |
| Illiteracy or primary school | 0.88 (0.77,0.98) |  | 0.99 (0.98,1.00) |  | 1.00 (1.00,1.00) |  |
| Secondary school | 0.87 (0.82,0.92) |  | 0.97 (0.94,0.99) |  | 0.98 (0.97,1.00) |  |
| High school or technical secondary school | 0.82 (0.74,0.90) |  | 0.92 (0.84,0.99) |  | 0.96 (0.88,1.00) |  |
| University degree and above | 0.84 (0.76,0.91) |  | 0.96 (0.93,1.00) |  | 0.97 (0.93,1.00) |  |
| **Occupation** |  | 0.035^*^ |  | 0.020^*^ |  | 0.116 |
| Working | 0.88 (0.79,0.97) |  | 0.96 (0.87,1.00) |  | 0.96 (0.87,1.00) |  |
| Retired | 0.84 (0.80,0.87) |  | 0.95 (0.94,0.97) |  | 0.98 (0.97,0.99) |  |
| **Marital status** |  | 0.488 |  | 0.949 |  | 0.424 |
| Married | 0.85 (0.81,0.88) |  | 0.95 (0.93,0.98) |  | 0.97 (0.95,1.00) |  |
| Unmarried | 0.91 (0.71,1.00) |  | 0.96 (0.80,1.00) |  | 1.00 (1.00,1.00) |  |
| **Monthly income(Chinese Yuan, CNY)** |  | 0.185 |  | 0.850 |  | 0.090 |
| ≤4000 | 0.82 (0.76,0.88) |  | 0.95 (0.90,0.99) |  | 0.96 (0.91,1.00) |  |
| >4000 | 0.88 (0.84,0.92) |  | 0.96 (0.94,0.98) |  | 0.99 (0.99,1.00) |  |
| **Smoking** |  | 0.668 |  | 0.907 |  | 0.037^*^ |
| Yes | 0.83 (0.73,0.92) |  | 0.96 (0.92,1.00) |  | 0.97 (0.94,1.00) |  |
| No | 0.85 (0.82,0.89) |  | 0.95 (0.93,0.98) |  | 0.98 (0.95,1.00) |  |
| **Drinking** |  | 0.833 |  | 0.483 |  | 0.195 |
| Yes | 0.84 (0.74,0.93) |  | 0.97 (0.93,1.00) |  | 0.98 (0.96,1.00) |  |
| No | 0.85 (0.82,0.89) |  | 0.95 (0.92,0.98) |  | 0.97 (0.95,1.00) |  |
| **Exercise** |  | 0.437 |  | 0.098 |  | 0.385 |
| Yes | 0.85 (0.81,0.89) |  | 0.95 (0.92,0.97) |  | 0.98 (0.96,1.00) |  |
| No | 0.85 (0.80,0.90) |  | 0.96 (0.92,0.99) |  | 0.97 (0.94,1.00) |  |
| **CHD type** |  | 0.218 |  | 0.112 |  | 0.030^*^ |
| Unstable angina | 0.86 (0.83,0.89) |  | 0.96 (0.95,0.98) |  | 0.99 (0.98,0.99) |  |
| Acute myocardial infarction | 0.88 (0.79,0.97) |  | 0.97 (0.94,1.00) |  | 0.99 (0.98,1.00) |  |
| **Duration of CHD (months)** |  | 0.333 |  | 0.308 |  | 0.391 |
| ≤0.5 | 0.85 (0.78,0.91) |  | 0.94 (0.88,1.00) |  | 0.95 (0.89,1.00) |  |
| 0.51-1 | 0.89 (0.86,0.93) |  | 0.97 (0.95,1.00) |  | 0.99 (0.98,1.00) |  |
| >1 | 0.82 (0.76,0.88) |  | 0.96 (0.93,0.98) |  | 0.99 (0.98,1.00) |  |
| **Disease state** |  | 0.789 |  | 0.374 |  | 0.550 |
| First episode | 0.85 (0.80,0.90) |  | 0.95(0.91,0.99) |  | 0.97 (0.92,1.00) |  |
| Relapse | 0.85 (0.81,0.90) |  | 0.96(0.94,0.98) |  | 0.99 (0.97,1.00) |  |
| **Comorbidities with Hypertension** |  | 0.238 |  | 0.169 |  | 0.399 |
| Yes | 0.83 (0.78,0.88) |  | 0.94 (0.91,0.98) |  | 0.97 (0.94,1.00) |  |
| No | 0.89 (0.85,0.93) |  | 0.98 (0.96,1.00) |  | 0.99 (0.97,1.00) |  |
| **Comorbidities with Diabetes** |  | 0.716 |  | 0.850 |  | 0.470 |
| Yes | 0.82 (0.75,0.90) |  | 0.93 (0.87,1.00) |  | 0.96 (0.90,1.00) |  |
| No | 0.87 (0.83,0.90) |  | 0.97 (0.95,0.98) |  | 0.99 (0.97,1.00) |  |
| **Comorbidities with Hyperlipidemia** |  | 0.503 |  | 0.200 |  | 0.674 |
| Yes | 0.84 (0.78,0.90) |  | 0.98 (0.96,1.00) |  | 0.99 (0.98,1.00) |  |
| No | 0.85 (0.81,0.90) |  | 0.94 (0.91,0.98) |  | 0.97 (0.94,1.00) |  |
| **Subjective well-being** |  | 0.017^*^ |  | 0.008^*^ |  | 0.002^*^ |
| WHO-5 scores <13 | 0.68 (0.45,0.92) |  | 0.81 (0.55,1.00) |  | 0.85 (0.57,1.00) |  |
| WHO-5 scores≥13 | 0.87 (0.84,0.90) |  | 0.97 (0.96,0.98) |  | 0.99 (0.98,1.00) |  |

^*^ *P* <0.05.
